# IgGs-Abzymes from the Sera of Patients with Multiple Sclerosis Recognize and Hydrolyze miRNAs

**DOI:** 10.3390/ijms22062812

**Published:** 2021-03-10

**Authors:** Evgeny A. Ermakov, Evelina M. Kabirova, Valentina N. Buneva, Georgy A. Nevinsky

**Affiliations:** Institute of Chemical Biology and Fundamental Medicine, Siberian Division of Russian Academy of Sciences, 8 Lavrentiev Ave, 630090 Novosibirsk, Russia; evgeny_ermakov@mail.ru (E.A.E.); kabevelun@gmail.com (E.M.K.); buneva@niboch.nsc.ru (V.N.B.)

**Keywords:** multiple sclerosis, abzymes, miRNA recognition and hydrolysis, autoimmune reactions

## Abstract

Autoantibodies-abzymes hydrolyzing DNA, myelin basic protein, and oligosaccharides have been revealed in the sera of patients with multiple sclerosis (MS). In MS, specific microRNAs are found in blood and cerebrospinal fluid, which are characterized by increased expression. Autoantibodies, specifically hydrolyzing four different miRNAs, were first detected in the blood of schizophrenia patients. Here, we present the first evidence that 23 IgG antibodies of MS patients effectively recognize and hydrolyze four neuroregulatory miRNAs (miR-137, miR-9-5p, miR-219-2-3p, and miR-219-5p) and four immunoregulatory miRNAs (miR-21-3p, miR-146a-3p, miR-155-5p, and miR-326). Several known criteria were checked to show that the recognition and hydrolysis of miRNAs is an intrinsic property of MS IgGs. The hydrolysis of all miRNAs is mostly site-specific. The major and moderate sites of the hydrolysis of each miRNA for most of the IgG preparations coincided; however, some of them showed other specific sites of splitting. Several individual IgGs hydrolyzed some miRNAs almost nonspecifically at nearly all internucleoside bonds or demonstrated a combination of site-specific and nonspecific splitting. Maximum average relative activity (RA) was observed in the hydrolysis of miR-155-5p for IgGs of patients of two types of MS—clinically isolated syndrome and relapsing-remitting MS—but was also high for patients with primary progressive and secondary progressive MS. Differences between RAs of IgGs of four groups of MS patients and healthy donors were statistically significant (*p* < 0.015). There was a tendency of decreasing efficiency of hydrolysis of all eight miRNAs during remission compared with the exacerbation of the disease.

## 1. Introduction

Multiple sclerosis (MS) is a chronic neuroimmune disease, the pathogenesis of which is characterized by the formation of foci of demyelination (plaques) of the gray and white matter of the brain and spinal cord, with subsequent neurodegeneration leading to brain atrophy [1,2]. The etiology of MS still remains unclear, and the most accepted pathogenesis theory assigns the main role in the destruction of the axon myelin-proteolipid shell to inflammation associated with important autoimmune reactions ([3], and the references therein). It is believed that several pathogens may play a role in the development of MS, including bacteria (such as *Chlamydia pneumoniae*, *Staphylococcus*, and *Mycoplasma pneumoniae*) producing different superantigens as well as some viruses (human herpesvirus, Epstein-Barr virus, and human endogenous retroviruses) (for reviews, see [3,4] and the references therein). It is known that after infection with viruses and bacteria, at first, there may be an accumulation of Abs against their components, which may have structural similarities with the components of blood and human cells [4,5]. Then, due to the mimicry of several specific proteins of viruses and bacteria with those of humans, epitope spreading and bystander activation failure of the immune system can occur, leading to the generation of Abs against own components of the human body and, as a result, to the development of MS.

In the last few years, artificial abzymes (Abzs; catalytic Abs to transition state analogs of chemical reactions) and natural Abzs have attracted much interest (reviewed in [6,7,8,9,10,11]). Similar to artificial Abzs against such analogs [6], natural abzymes in different mammals can be produced directly against enzyme substrates, acting as haptens, simulating the transition states of chemical reactions [6,7,8,9,10,11]. Anti-idiotypic second antibodies against the active centers of various enzymes can also possess catalytic activities [6,7,8,9,10,11]. Natural Abzs-splitting oligopeptides, proteins, DNA, RNA, nucleotides, and polysaccharides have been revealed in sera of patients suffering from several autoimmune and viral diseases (systemic lupus erythematosus (SLE), MS, Hashimoto thyroiditis, polyarthritis, tick-borne encephalitis, and HIV-infected patients) [6,7,8,9,10,11]. Healthy humans do not develop Abzs with detectable enzymatic activities; their activities usually absent or on the borderline of the detection methods’ sensitivity [6,7,8,9,10,11]. Nevertheless, germline Abs of healthy people sometimes can possess high-level promiscuous, amyloid- or superantigen-directed activities [12].

The statistically significant detection of Abzs is possible at the earliest stages of various autoimmune diseases (AIDs), when changes in Abs titers to specific antigens of various pathologies still correspond to the range changes in the titers of these Abs in healthy donors [6,7,8,9,10,11]. According to modern data, the abzymes’ presence in the blood is a clear sign of the beginning and following progress of autoimmune processes in humans and mammals.

It has been shown that myelin basic protein (MBP)- [13,14,15,16], DNA- [17,18], oligosaccharide- [19], and histone-hydrolyzing [20] as well as redox [21] enzymatic activities are intrinsic properties of antibodies from the sera of MS patients. The same catalytic activities have been found in the sera of SLE patients [11,21,22,23,24,25].

Abzs with MBP-hydrolyzing activity can attack MBP in the myelin-proteolipid sheath of axons and, therefore, may play a very negative role in MS pathogenesis [10]. Abzs with DNase activity are cytotoxic, can penetrate the cell nuclear, cause fragmentation of DNA, and induce cell death by apoptosis [26,27,28]. We have recently shown that the specific enzymatic activities of Abs from the CSF of MS patients, hydrolyzing DNA, MBP, and oligosaccharides, are about 30–60-fold higher than from the blood serum of the same patients [29,30,31].

Another established indicator of the beginning and development of AIDs is abzymes hydrolyzing RNA of human blood. The same polyclonal Abs preparations from sera of SLE patients hydrolyzed RNA approximately 30–300 times faster than DNA [32,33]. Abzs with RNase activity in AIDs, therefore, are of particular interest.

It was shown that MS is a multifactorial disease, the pathogenesis of which could be attributed to many different factors [34]. MiRNA is a class of small (approximately 22–25 nucleotides) noncoding RNAs involved in the post-transcriptional regulation of expression of many genes [35,36] and the regulation of transcription and neuroinflammation [35,36,37]. In MS and SLE, specific miRNAs are found in blood and cerebrospinal fluid, which are characterized by the increased expression [37,38,39,40]. The extracellular functions of miRNAs include signaling between cells and the regulation of angiogenesis, neurogenesis, and cell proliferation [41]. The change in miRNA expression in the extracellular compartments, in many cases, is associated with pathological processes. For example, as a result of inflammatory processes in MS and SLE, transcription, processing, or maturation of specific miRNAs may be changed. Such miRNAs are considered biomarkers of these diseases [42]. Therefore, in the research of additional important factors of understanding the pathogenesis of MS, the possible role of miRNAs may also be important. In addition, it is possible that antibodies and abzymes against miRNA can also play an important role in the pathogenesis of MS.

For the first time, abzymes that specifically hydrolyze some miRNAs were found in the blood of schizophrenia patients [43,44]. Hydrolysis of miR-137, miR-9-5p, miR-219-2-3p, and miR-219a-5p, which play an important role in the regulation of the functioning of several genes in patients with schizophrenia, was analyzed. The recognition and site-specific hydrolysis of these miRNAs by IgGs were revealed. These data show that in some patients with schizophrenia, the appearance of typical autoimmune processes and gene dysfunctions due to miRNA hydrolysis can occur [43,44]. It is believed that schizophrenia and multiple sclerosis are very different diseases. There is no doubt that the medical manifestations of these two diseases are very different. At the same time, these diseases characterize several very similar biochemical and immunological indicators. As mentioned above, the presence of abzymes in the blood is a clear and statistically significant indicator of the onset and progress of autoimmune processes in humans and mammals [6,7,8,9,10,11]. Abzyme-antibodies that hydrolyze DNA, RNA, MBP, five histones, and oligosaccharides were found in the blood and CSF of MS patients [10,13,14,15,16,17,18,19,20,29,30,31]. The blood of patients with schizophrenia also contains antibodies-abzymes with DNase, protease (hydrolysis of MBP and five histones), and amylase activities [43,44,45,46,47]. This indicates some similarities of autoimmune processes in patients with MS and schizophrenia.

As mentioned above, miRNAs have important functions in living organisms. Therein, the same miRNAs were found to be important in schizophrenia [48,49,50,51,52,53,54,55], MS [56,57,58], and systemic lupus erythematosus [40,41,42]. miR-137 is responsible for the proliferation and differentiation of embryonic and neurons stem cells, as well as the maturation of the synapses [59]; it inhibits AMPA-receptor-mediated synaptic transmission by reduction of the expression of the GluA1 protein of this receptor [60] and disrupts synaptic plasticity [61]. In the regulation of dopamine, D2 receptor expression miR-9-5p is involved, whereas miR-206 is associated in the site-specific regulation of NT5C2 [62]. MiR-9-5p participates in neuronal migration, and its expression is reduced in neuronal cell precursors [63]. miR-219 is important for the differentiation of oligodendrocytes and the myelination of axons of neuronal cells [64]. Proinflammatory miR-155 and miR-326 and anti-inflammatory miR-21 and miR-146a were upregulated in both brain white matter lesions and peripheral blood mononuclear cells from MS patients [56]. Moreover, the levels of miR-21 and miR-146a were increased in CSF and active brain lesions of MS patients [58]. Moreover, the expression levels of miR-146a and miR-155 in monocytes of relapsing–remitting and progressive forms of MS patients are significantly different [57]. Therefore, the destruction of miRNAs due to various factors can contribute to greater dysregulation of miRNAs in MS.

Overall, the sera of schizophrenia, MS, and SLE patients contain several common neuroregulatory miRNAs (miR-137, miR-9-5p, miR-219-2-3p, miR-219-5p), whereas in immunoregulation, the sera contain miR-21-3p, miR-146a-3p, miR-155-5p, miR-326, and some other microRNAs [37,48,49,50,51,52,53,54,55,56,57,58,59,60,61,62,63,64,65]. In addition, it was shown recently that IgGs from the blood of SLE patients also efficiently hydrolyzed these eight microRNAs [66]. Such similarity in the increase in the expression of these regulatory miRNAs in patients with MS, SLE, and schizophrenia may lead to the appearance of some common features in these diseases. Moreover, some similar common neuropsychiatric indicators of schizophrenia have been revealed in 40–50% of multiple sclerosis and SLE patients [3].

Therefore, it is interesting to elicit if the abzymes recognizing and hydrolyzing miRNAs that have been found in schizophrenia and SLE patients can exist in patients with multiple sclerosis and how much they may differ or be similar.

Taking into account the ability of Abs to hydrolyze RNA, together with the important role of miRNAs in the proliferation, differentiation, and maturation of neuronal cells and the relationship of miRNAs with the development of multiple sclerosis, in this work, we study the miRNA recognition and hydrolysis by IgGs of MS patients. In addition, we compare substrate specificity antibodies in the hydrolysis of miRNAs specific to MS.

## 2. Results

### 2.1. Characteristic of Patients

It was earlier shown in several articles that Abs from sera of healthy humans, with the exception of rare IgGs, usually could not hydrolyze polymeric RNAs [67,68,69,70]. Here, we have analyzed the recognition of various miRNAs and their hydrolysis by 23 IgGs of MS patients and 14 healthy donors. The characteristics of 23 MS patients of four different groups—clinically isolated syndrome of MS (CISMS, eight patients); relapsing–remitting MS (RRMS, nine patients); secondary progressive MS (SPMS, five patients); primary progressive MS (PPMS; 1 patient)—are given in the Supplementary Materials (Table S1).

### 2.2. Purification and Characterizing of IgGs

**Figure 1:**
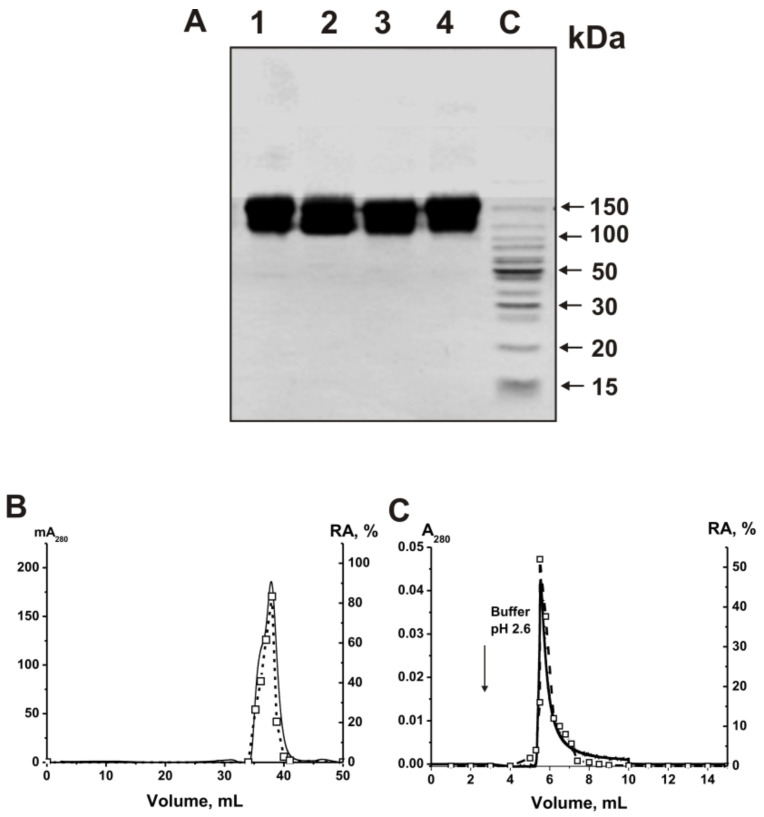
SDS-PAGE analysis of the electrophoretic homogeneity of ms-IgG1 (Lane 1), ms-IgG_mix_ (multiple sclerosis (MS) patients; Lane 2), healthy-IgG1 (Lane 3), and healthy-IgG_mix_ (healthy donors; Lane 4) in 4–18% gradient gel, followed by silver staining (15 µg IgGs were used). (**A**) Ms-IgG1 and healthy-IgG1 correspond to individual antibodies. The arrows (Lane C) indicate the positions of protein molecular mass markers. Ms-IgG_mix_ FPLC gel filtration on a Superdex 200 column equilibrated with acidic buffer (pH 2.6) after Abs preincubation using this buffer (**B**), and affinity chromatography of the ms-IgG_mix_ on anti-IgG-sepharose bearing mouse Abs against human IgGs (**C**). (—), absorbance at 280 nm (A_280_); (□), RAs (%) of ms-IgG_mix_ in the hydrolysis of RNA (**B**,**C**). Complete hydrolysis of miR-326 for 7 h using 5 µl of the eluate was taken for 100% (**B**,**C**). The error from two experiments in the initial rate determination in each case did not exceed 7–10%.

Electrophoretically homogeneous IgG antibodies were isolated from the sera of 14 healthy volunteers and 23 MS patients by affinity chromatography of serum proteins on protein A-sepharose in terms of deleting nonspecifically bound proteins, followed by using FPLC gel filtration in acidic conditions (pH 2.6), destroying immune complexes, according to [13,14,15,16,17]. The mixtures of equal milligrams of polyclonal IgGs of 23 MS patients (ms-IgG_mix_) and 14 healthy donors (healthy-IgG_mix_) were prepared. The electrophoretic homogeneity of the typical 150-kDa IgG_mix_ preparations was confirmed by SDS-PAGE with silver staining only one protein band was revealed (Figure 1).

### 2.3. Application of Strict Criteria

To prove that the ribonuclease activity belongs to the IgGs of MS patients, four strict previously developed criteria were used [6,7,8,9,10,11,71]. They are summarized as follows: (a) ms-IgG_mix_ and healthy-IgG_mix_ (corresponding to central parts of the peaks after gel filtration) were electrophoretically homogeneous (Figure 1A); (b) ms-IgG_mix_ gel filtrated in the buffer (pH 2.6), destroying strong noncovalent complexes, did not lose ribonuclease activity, and the RNase activity peak coincided with the peak of intact antibodies (Figure 1B); (c) anti-IgG-sepharose completely bind the RNase activity and the peaks of this activity and IgGs coincide at their specific elution by acidic buffer (pH 2.6) (Figure 1C).

Canonical human ribonucleases have significantly lower molecular weights (13–15 kDa) than intact IgGs (150 kDa). Therefore, the coincidences of the peaks of ribonuclease activity and IgGs give direct evidence that MS IgGs cleavage RNAs and are not contaminated by canonical ribonucleases.

**Figure 2:**
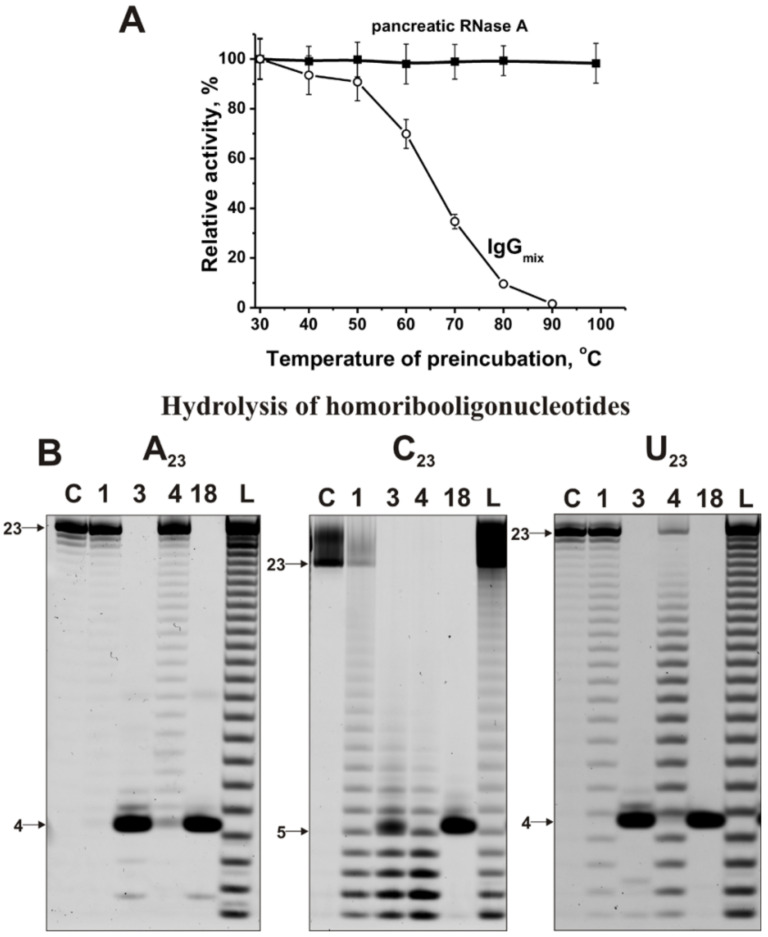
Comparison of thermal stability of IgG_mix_ and human RNase A (**A**). IgG_mix_ and RNase A were incubated for 15 min at different temperatures, and then their relative RNase activities were estimated using miR-137. The patterns of 5’-Flu-(pA)_23_, 5’-Flu-(pC)_23_, and 5’-Flu-(pU)_23_ (0.01 mg/mL) splitting by IgGs (0.6 µM IgGs; 1 h of incubation) from sera of four different MS patients (**B**). The products of the cleavage were detected due to the fluorescent residue (Flu) on their 5′-ends. The lengths of the products, numbers of antibodies, and homo-oligonucleotides used are indicated in panel (**B**).

In addition, all canonical ribonucleases are known as very thermostable enzymes, and antibodies are much less thermostable molecules. As can be seen from Figure 2A, after preincubation of RNase A for 15 min, even at 100 °C, it practically does not lose its activity, while the activity of IgG_mix_ is zero.

### 2.4. Estimation of Relative RNase Activity

RNase activity was estimated quantitatively using fluorescently labeled homo-oligonucleotides (ONs) 5’-Flu-(pA)_23_, 5’-Flu-(pC)_23_, and 5’-Flu-(pU)_23_ and eight miRNAs. Figure 2B demonstrates the hydrolysis of these homo-ONs. One can see that various IgGs hydrolyze (pA)_23_, (pC)_23_, and (pU)_23_ in different ways. The recognition of all homo-oligonucleotides occurs mainly nonspecifically. Therefore, the hydrolysis of (pA)_23_ proceeds mainly nonspecifically at nearly all internucleoside bonds but with the predominant formation of pentanucleotides (Figure 2B). A similar situation of nonspecific recognition and hydrolysis, leading to predominant formation of pentanucleotides, is observed for (pC)_23_, but in this case, different products from mono- to tetranucleotides are also formed (Figure 2B). The hydrolysis of (pU)_23_ is more similar to the hydrolysis of (pA)_23_ (Figure 2B)_._ Altogether, IgGs recognize homo-oligonucleotides, and the hydrolysis of all three homo-ONs occurs predominantly nonspecifically. However, pentanucleotides are major products of hydrolysis in the case of most IgG preparations and homo-oligonucleotides (ONs).

**Figure 3:**
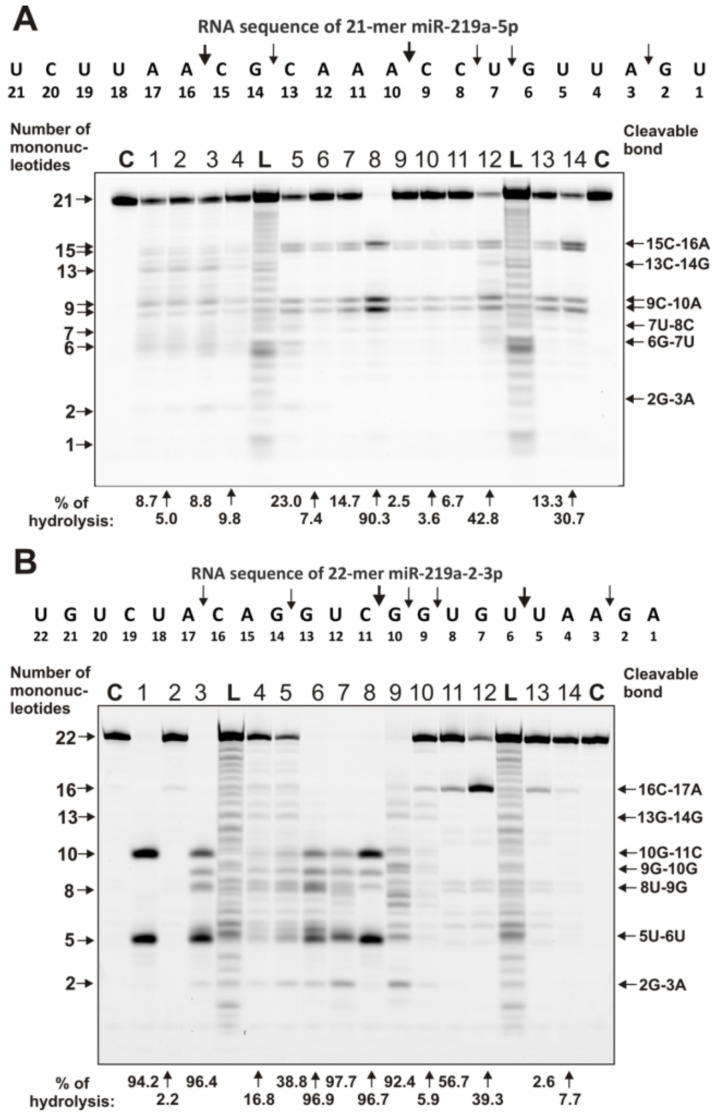
The patterns of cleavage of oligonucleotides Flu-miR-219-2-5p (**A**) and Flu-miR-219-2-3p (**B**) by IgGs (0.6 µM IgGs; 1 h of incubation) from sera of 14 different MS patients. The products of splitting were detected due to the fluorescent residue (Flu) on the 5′-ends of the oligonucleotides. Lane C corresponds to ONs incubated in the absence of Abs, whereas Lanes L corresponds to the length of oligonucleotide markers. The numbers of IgG preparations, lengths of the products, and the percentage of microRNA cleavage by each IgG preparation are shown in panels (**A**,**B**).

**Figure 4:**
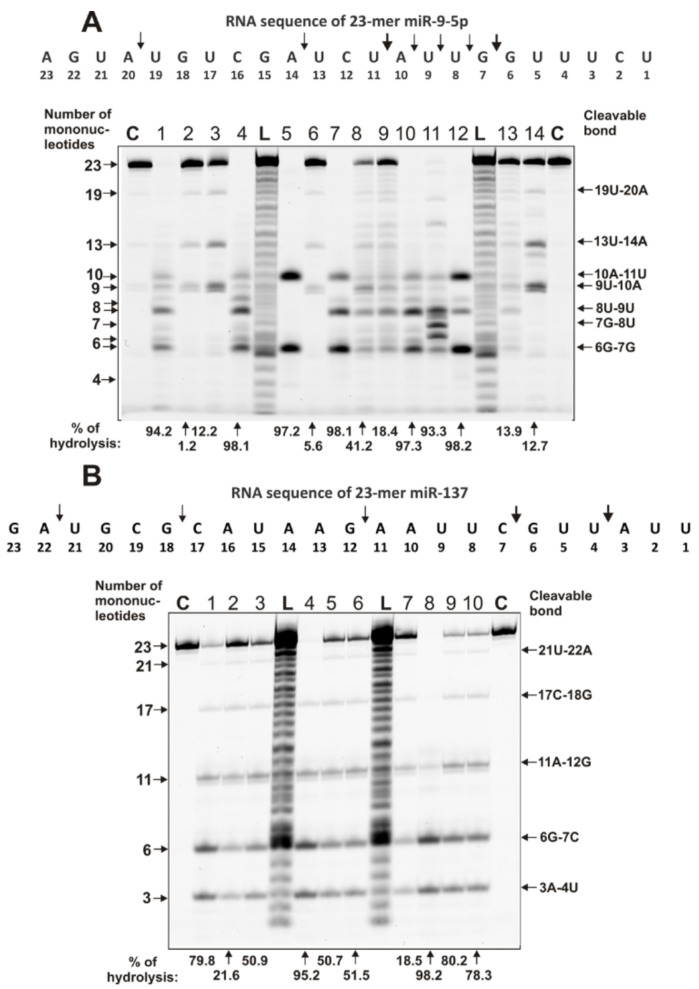
The patterns of cleavage of oligonucleotides Flu-miR-9-5p (**A**) and Flu- miR-137 (**B**) by different preparations of 0.6 µM IgGs for 1 h of incubation from sera of 10–14 different MS patients. The products of hydrolysis were detected due to the fluorescent residue (Flu) on the 5’-ends of the miRNAs. Lane C corresponds to ONs incubated in the absence of Abs, while Lane L corresponds to the length of ONs. The numbers of IgG preparations, lengths of the products, and the percentage of microRNA cleavage by each preparation of IgG are indicated in panels (**A**,**B**).

Unlike homo-ONs, the recognition and hydrolysis of all eight miRNAs are predominantly site-specific. Figure 3 and Figure 4 demonstrate patterns of the hydrolysis of four neuroregulatory miRNAs: miR-219-5p, miR-219-2-3p, miR-9-5p, and miR-137. Typical examples of the hydrolysis of miR-219-5p by 14 of the 23 IgG preparations are shown in Figure 3A

It can be seen that all IgGs, due to specific recognition, hydrolyze this miRNA mainly at two major sites: 15C-16A and 9C-10A; only some of them hydrolyze RNA more effectively in the 13G–14C site than in the neighboring 15C–16A site. For all 23 IgG preparations, the same type of site-specific hydrolysis of miR-219-5p was observed.

A completely different situation was revealed in the hydrolysis of miR-219-2-3p (Figure 3B). Some IgGs show very poor efficacy in this miRNA hydrolysis. For example, IgG2, IgG13, and IgG14 hydrolyzed this miRNA very weakly and only in the 16C–17A site (Figure 3B). At the same time, some IgGs hydrolyzed miR-219-2-3p nonspecifically at almost all internucleoside phosphate groups, with comparable efficacy (for example, IgG4, IgG5, IgG9, and IgG10). Some IgGs split this miRNA at several sites located between two major sites of the hydrolysis: 10G–11C and 5U–6U. Interestingly, IgG1 specifically recognized and hydrolyzed this miRNA strictly at only two sites: 10G–11C and 5U–6U (Figure 3B). Such a difference in the efficiency of hydrolysis of miR-219-2-3p by IgGs from the blood of various MS patients may be due to the fact that in some of them, the formation of Abs and abzymes that are specifically against miR-219-2-3p is ineffective or almost does not occur. As shown earlier, individual DNA and RNA have weak immunogenicity, which sharply increases when their complexes with proteins are formed [8,9,10,11]. It is possible that the level of immunogenicity of some microRNAs is lowered.

A variety of patterns of miR-9-5p hydrolysis by IgGs from the blood of different patients with MS was observed (Figure 4A).

Some IgGs weakly split this miRNA, mainly in major sites of hydrolysis (13U–14A, 10A–11U, and 6G–7G). However, most of the IgGs hydrolyze miR-9-5p with different efficacy across all sites between the major ones: 10A–11U and 6G–7G. IgG5 hydrolyzed miR-9-5p only in these two major sites.

Interestingly, all IgGs recognize and hydrolyze miR-137 very specifically only at five major sites: 6G–7C > 3A-4U > 11A-12G > 17C-18G > 21U-22A (Figure 4B).

**Figure 5:**
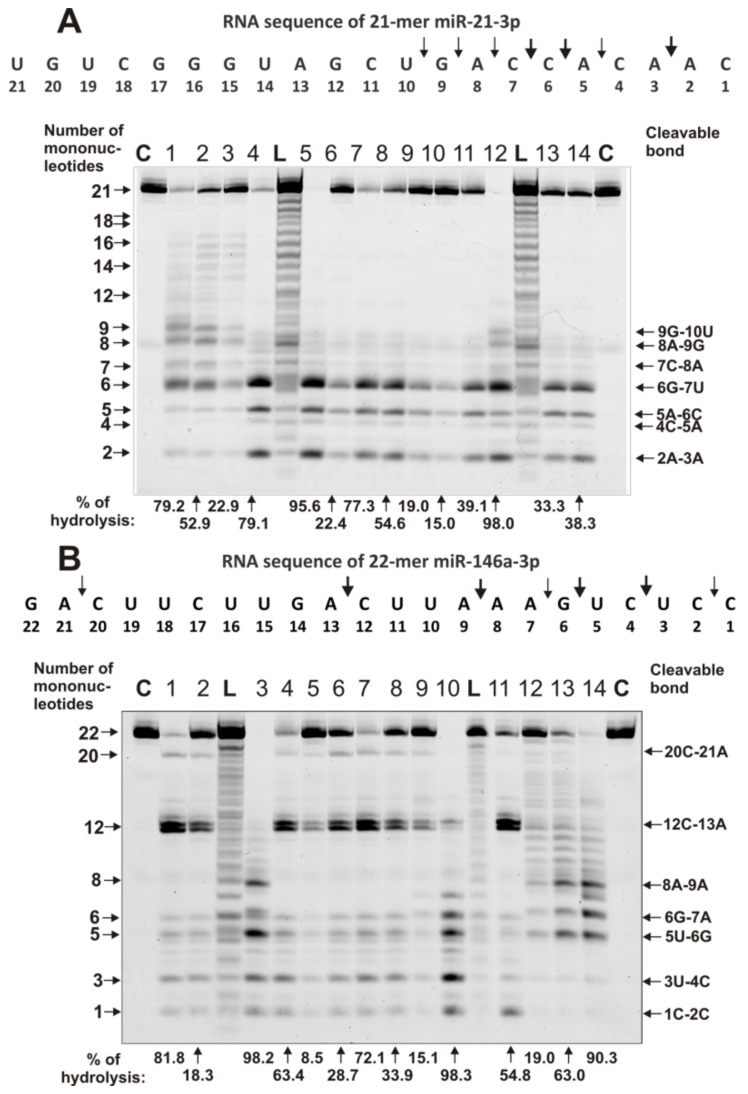
The patterns of hydrolysis of oligonucleotides Flu-miR-21-3p (**A**) and Flu-miR-146a-3p (**B**) by preparations of 0.6 µM IgGs for 1 h of incubation from sera of 14 different MS patients. The products of the cleavage were detected due to the fluorescent residue (Flu) on the 5’-ends of the oligonucleotides. Lane C corresponds to ONs incubated in the absence of Abs, whereas Lane L corresponds to lengths of oligonucleotide markers. The lengths of the products, numbers of IgGs, and the percentage of microRNA hydrolysis by each IgG preparation are shown in panels (**A**,**B**).

**Figure 6:**
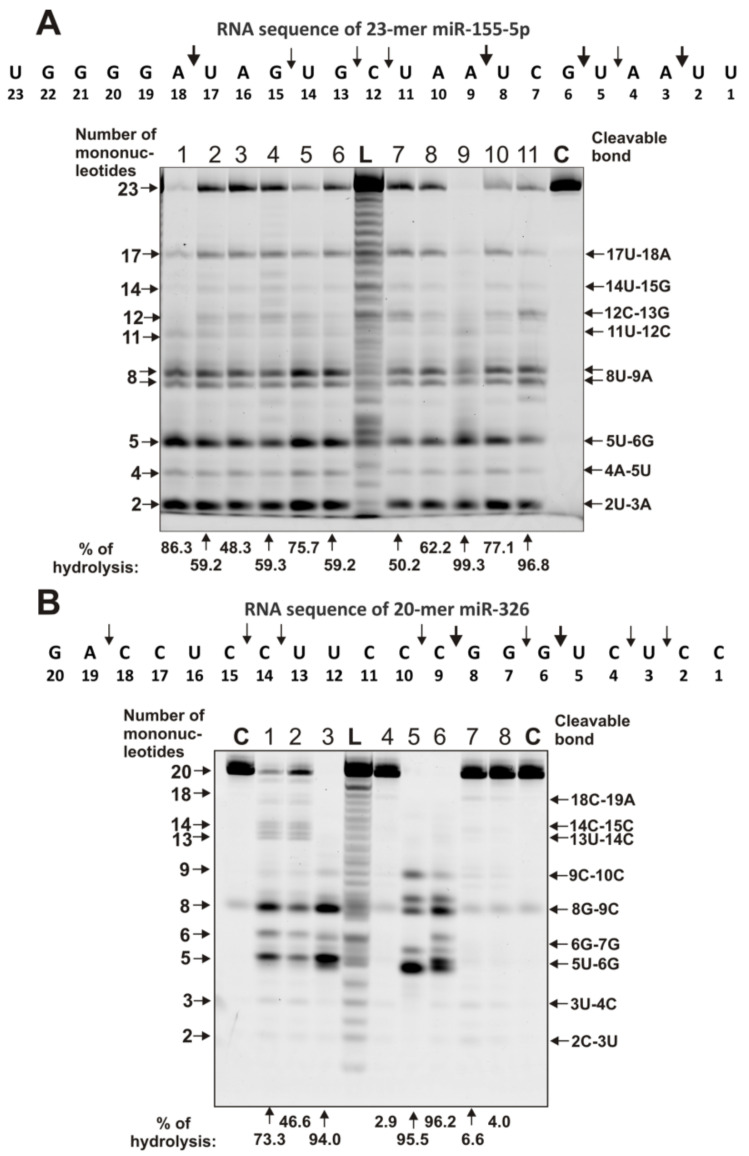
The patterns of splitting of oligonucleotides Flu-miR-155-5p (**A**) and Flu- miR-326 (**B**) by preparations of 0.6 µM IgGs for 1 h of incubation from sera of 8 and 14 different MS patients. The products of hydrolysis were detected due to the fluorescent residue (Flu) on the 5′-ends of the miRNAs. Lane C corresponds to ONs incubated in the absence of IgGs, whereas Lane L corresponds to control oligonucleotide length markers. The numbers of IgG preparation, lengths of the products, and the percentage of microRNA cleavage by each IgG preparation are shown in panels (**A**,**B**).

Several typical examples of 4 immunoregulatory miRNAs’ hydrolysis are given in Figure 5 and Figure 6. IgG1–IgG3 showed combined nonspecific and site-specific recognition and the hydrolysis of miR-21-3p (Figure 5A).

However, most of the IgGs hydrolyzed this miRNA with different efficacy across all sites, from 9G-10U to 2A-3A, among which there are three major sites: 6G-7U, 5A-6C, and 2A-3A (Figure 5A). Approximately the same situation was observed for miR-146a-3p (Figure 5B). Several IgG preparations (IgG12-IgG14) showed a combination of specific and nonspecific cleavage of this miRNA. Mainly, most of the IgGs split this miRNA specifically in sites from 8A-9A to 1C-2C; 12C-13A was the best site of hydrolysis (Figure 5B).

The splitting of miR-155-5p by all IgGs occurred mainly in several sites from 17U-18A to 2U-3A, among which several major ones were pronounced: 17U-18A, 8U-9A, 5U-6G, and 2U-3A (Figure 6A).

Some IgGs weakly hydrolyzed miR-326, while others were more efficient, but in all cases, the same major cleavage sites were observed: 8G-9C and 5U-6G (Figure 6B). Above, below, and between these sites, there are also sites of moderate and weak hydrolysis.

Several IgG preparations were estimated as to their affinity for several microRNAs (miR-137, miR-9-5p, miR-219-2-3p, and miR-219a-5p) in terms of apparent *K*_m_ values for miRNAs. Interestingly, the *K*_m_ values turned out to be comparable for all miRNAs and varied from 1.6 to 4.6 µM.

### 2.5. Comparison of Relative Activities in the Hydrolysis of RNAs with Abzymes from the Blood of Different Patients

The relative activities of 23 individual IgGs of MS patients were significantly different. However, all 23 preparations had RNase activity in the hydrolysis of all miRNAs. It was previously shown that the blood of carefully selected, conditionally healthy donors does not contain IgGs hydrolyzing DNA and RNA [17,18,32,33,67,68,69,70]. However, at present, there has been a change in the diet of people, and the level of environmental pollution has increased. Therefore, it turned out to be difficult to find the blood of really healthy donors. In this work, 14 preparations from the blood of conditionally healthy donors were used for control. Some of them showed weak or very weak but reliably detectable RNase activity (see below).

The relative activities (RAs) in the hydrolysis of eight miRNAs by IgGs from MS patients and conditionally healthy donors were measured and normalized to standard conditions. Then, all 23 IgG preparations were divided into 4 groups depending on the type of MS pathology. Averaged values of RNase activity (three independent experiments in the case of each patient) in the hydrolysis of eight miRNAs by IgGs from the blood of patients with the clinically isolated syndrome of MS (CISMS; all detailed data are given in Table S2), relapsing–remitting MS (RRMS; Table S3), primary progressive (PPMS) and secondary progressive MS (SPMS; Table S4), and conditionally healthy donors (Table S5) are given. Some sets of values did not match the normal Gaussian distribution. Therefore, not only the average values ± SD but also the median (M) and interquartile ranges (IQRs) were estimated for each group of patients and conditionally healthy donors (Tables S2–S5). The mean values, median (M), and interquartile ranges (IQRs) for all groups of patients are summarized in Table 1.

Average relative activities (RAs) in the hydrolysis (%) of various miRNAs by IgGs of these four groups of MS patients and conditionally healthy donors decreased in different orders; the detailed data are given in Table S6, and the main data on the difference in the relative rate of hydrolysis of eight microRNAs by various IgGs are shown in Table 2. All average relative activities for the five groups and eight microRNAs were estimated in relative units from **1** (maximum activity) to **8** (minimum activity). Maximum average RAs (**1**) were observed for miR-155-5p in the case of two IgGs groups: CISMS and RRMS. However, even in the case of other groups, RAs characterizing the hydrolysis of miR-155-5p were also high (Table 2). The minimum RA values (**8**) in the case of the four IgG groups were observed for various miRNAs: CISMS (miR-219-5p and miR-219-2-3p), RRMS and PPMS (miR-137), and SPMS (miR-326). For all five IgG groups, the average relative indices in the hydrolysis of individual eight microRNAs were calculated, and they decrease in the following order (average relative units): miR-155-5p (**2**), miR-21-3p (**3.6**), miR-9-5p (**4**), miR-146a-3p (**4**), miR-219-5p (**5.0**), miR-219-2-3p (**5.2**), miR-326 (**5.6**), and miR-137 (**6.6**) (Table 2).

Three of the 14 IgG preparations from the blood of conditionally healthy donors did not show reliably tested activity in the hydrolysis of any of eight miRNAs (Table S5). The remaining 11 IgG preparations showed weak or very weak but reliably detectable hydrolysis activity from three to eight different miRNAs. However, all individual IgGs of healthy donors possessing RNase activity hydrolyzed different miRNAs (Table S5).

The relative values of average RAs for each group of antibodies (patients), corresponding to the hydrolysis of all eight RNAs, were also evaluated (Table 1). The relative average RA corresponding to the hydrolysis of eight RNAs by 14 IgGs from healthy donors (2.3 ± 2.7, M = 0.8, IQR = 4.4%), according to average values and medians, were significantly lower than those for MS patients (-fold): RRMS (24.4 and 41.9, respectively), CISMS (19.5 and 49.0), and SPMS (19.5 and 24.9). Overall, for all eight miRNAs, the average RA for conditionally healthy donors is 22.3–57.3-fold lower than that for all 23 MS patients.

First, using the Kruskal–Wallis ANOVA test, it was shown that the relative RAs in the hydrolysis of various miRNAs were statistically significantly (*p* < 0.026) different for all types of MS courses in the case of all miRNAs, except for miR-9-5p (*p* = 0.15). In addition, the same results on the significance of RA value differences for various groups of MS patients were obtained using the Mann–Whitney test. Differences between healthy donors and all groups of MS patients (CISMS, RRMS, SPMS, and PPMS) were also statistically significant; *p*-values varied from 0.0001 to 0.015.

### 2.6. Correlation Coefficients

Correlation coefficients (CCs) between values corresponding to RA sets of IgGs in the hydrolysis of eight miRNAs were calculated (see Tables S2–S5).

For the clinically isolated syndrome of MS, CCs were mostly positive and varied from +0.43 to +0.95 (Table S2). However, there were observed low positive CCs only for RAs in the case of miR-9-5p and miR-155-5p (+017), miR-146a-3p, and miR-155-5p (+0.12). Low negative CCs were revealed for miR-137 and miR-155-5p (-0.04) as well as miR-9-5p and miR-155-5p (-0.05). Thus, in these groups of MS patients, low positive and negative CCs were observed mainly for miR-155-5p.

CCs of RA sets appurtenant to the hydrolysis of the eight miRNAs, in the case of relapsing–remitting MS, were mostly positive and high (+0.36–+0.93) except for some of them: miR-155-5p and miR-326 (+0.21), miR-219-5p and miR-21-3p (+0.07), and miR-219-5p and miR-146a-3p (+0.14) (Table S3). For miR-137, low negative CCs with miR-219-5p (-0.13) and miR-326 (-0.31) were observed (Table S3). In the case of the hydrolysis of the eight miRNAs by IgGs of the secondary progressive MS group, all CCs were positive and high (+0.87 to +0.99) (Table S4).

### 2.7. The Activity of Antibodies of Patients with a Different Course of the Disease

**Figure 7:**
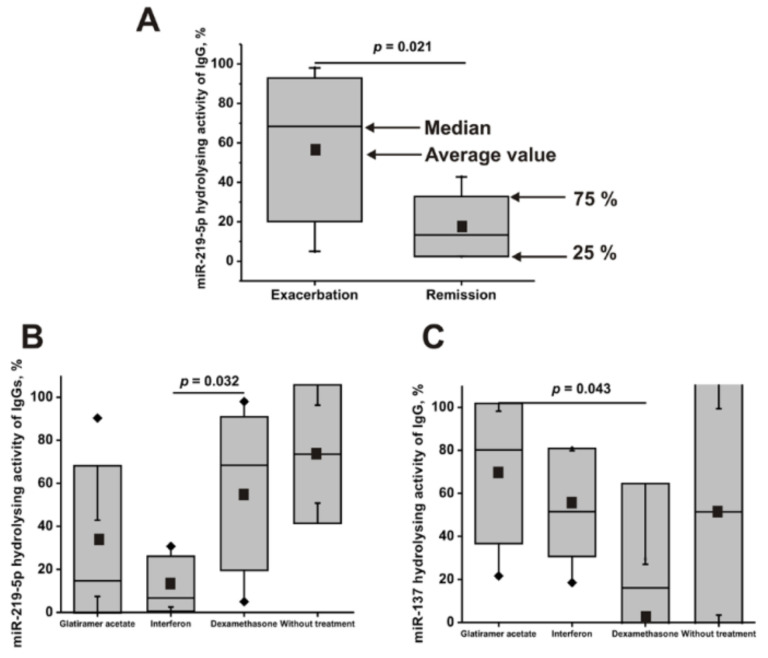
Comparison of the average values of relative activities (■), medians (^__^), and interquartile IQR ranges (gray areas) for groups of patients with exacerbation and relaxing courses of MS (**A**), as well as average activities of antibodies corresponding to groups of patients before (2 patients) and after their treatment with dexamethasone (10 patients), glatiramer acetate (5 patients), and interferon β-1b (5 patients) in the hydrolysis of miR-155-5p (**B**) and miR-137 (**C**).

First, all 23 patients with MS were divided into two groups corresponding to exacerbation (16 patients) and remission (7 patients) courses of the disease. The difference in mean values and medians (M) for patients with remission and exacerbation courses of the disease were statistically insignificant in the hydrolysis of miR-137 (*p* = 0.09), miR-9-5p (0.62), miR-219-2-3p (0.15), miR-21-3p (0.53), miR-146a-3p (0.82), miR-155-5p (0.27), and miR-326 (0.57). Only in the case of hydrolysis of miR-219-5p was statistically significant (*p* = 0.021) 3.2–5.1–fold lower values detected (average value 17.6 ± 15.5, M = 13.3, IQR = 5.5%) for remission compared with that for patients with the exacerbation course (average value 56.5 ± 36.3, M = 68.4, IQR = 13.5%) of the disease (Figure 7A). Nevertheless, in total, during remission, there is a tendency of decrease in the efficiency of hydrolysis of all eight miRNAs (average value = 44.7 ± 17.4; average M = 35.1 ± 26.8%) compared with the exacerbation of the disease (average value = 54.0 ± 10.2; M = 49.6 ± 21.5%).

### 2.8. The Activity of Antibodies of Patients Treated with Different Drugs

Then, all 23 patients with MS were divided into groups before (2 patients) and after patient treatment with dexamethasone (10 patients), glatiramer acetate (5 patients), and interferon β-1b (5 patients). For most of these groups of patients treated with different drugs, there was no significant difference in the relative RNase activity values corresponding to the hydrolysis of various miRNAs; all *p*-values varied from 0.098 to 0.95. Only two exceptions were found. The patients treated with interferon (average value = 13.3 ± 12.8; M = 6.7, IQR = 3.6%) in the hydrolysis of miR-219-5p demonstrated statistically significant 4.2–10.2-fold differences (*p* = 0.032) in comparison with those treated with dexamethasone (average value = 55.2 ± 35.7; M = 68.4, IQR = 18.6%) (Figure 7B).

In addition, patients treated with dexamethasone (average value = 29.5± 35.0; M = 16.1, IQR = 7.4%) showed, in the hydrolysis of miR-137, statistically significant (*p* = 0.043) 2.3–13.1-fold lower activity in the hydrolysis of miR-137 in comparison with those treated with glatiramer acetate (average value = 69.2 ± 32.6; M = 80.2, IQR = 50.9%) (Figure 7C).

Using the analysis of the Spearman correlation coefficient, a significant (*p* < 0.05) negative correlation was found between the hydrolysis level of miR-219-5p with age (CC = −0.55) and with the duration of the disease (CC = −0.53). Therefore, it can be assumed that with the increase of age and duration of the disease, the level of the miRNAs’ hydrolyzing activity may be, to some extent, reduced.

However, due to the difficulty of finding patients before treatment (only 2), we were not able to analyze the differences between MS patients before and after their treatment with different drugs.

## 3. Discussion

It has been shown recently that antibodies from the blood of schizophrenia [43,44] and SLE [66] patients effectively hydrolyze four individual miRNAs: miR-137, miR-9-5p, miR-219-2-3p, and miR-219a-5p. The blood of patients with MS and SLE contains abzymes that recognize and hydrolyze several of their own antigens: MBP, DNA, histones, and oligosaccharides [8,9,10,11,13,14,15,16,17,18,19,20]. In schizophrenia [37,38,39,48,49,50,51,52,53,54,55], SLE [40,41,42], and MS [56,57,58] patients, some specific miRNAs are characterized by increased expression and participate in signaling between cells, the regulation of angiogenesis, neurogenesis, and cell proliferation. The change in miRNA expression in the extracellular compartment is associated with specific pathological processes. In the case of all these three diseases, these microRNAs could potentially have a similar role. Possibly due to the similarity in microRNA and autoimmune processes, some similar common neuropsychiatric indicators of schizophrenia were revealed in 40–50% of multiple sclerosis and SLE patients [3].

It has been shown that all 23 MS IgGs effectively recognize and hydrolyze eight different miRNAs and three homo-oligonucleotides (Figure 2, Figure 3, Figure 4, Figure 5 and Figure 6, Table 1 and Tables S2–S6). The recognition and hydrolysis of all three homo-ONs occurred predominately nonspecifically, but pentanucleotides were the major products of their hydrolysis by most IgGs (Figure 2B). In contrast, the recognition and hydrolysis of eight miRNAs were mostly site-specific (Figure 3, Figure 4, Figure 5 and Figure 6). Nevertheless, some fractions of IgGs from sera of several patients, in parallel with specific splitting, recognized and hydrolyzed some miRNAs nonspecifically, similar to that for the hydrolysis of homo-ONs (for example, Figure 3, Lanes 4, 5, 9, and 10; miR-219a-2-3p). Several IgGs (for example, IgG1-IgG3) demonstrated a combination of specific and nonspecific recognition and cleavage of miR-21-3p (Figure 5A). However, most of the IgGs recognize and split this miRNA mainly and specifically in sites 6G-7U, 5A-6C, and 2A-3A. Several IgG preparations recognized and cleaved miRNA strictly only at two specific sites (for example, IgG1, miR-219a-2-3p Figure 3B; IgG5, miR-9-5p, Figure 4A). However, in most cases, several major cleavage sites were observed (Figure 3, Figure 4, Figure 5 and Figure 6). Overall, the number of major and moderate sites of recognition and hydrolysis was individual for each IgG preparation, miRNA, and IgG preparation (Figure 3, Figure 4, Figure 5 and Figure 6). Almost identical patterns, corresponding to the formation of four major products, were observed for all 23 IgGs in the case of miR-137 (Figure 4B). Most IgG cleave sites were specific for several different miRNAs (Figure 3, Figure 4, Figure 5 and Figure 6).

The question arises about a possible reason for the different ratios of relative site-specific and nonspecific recognition and cleavage sites of various miRNAs from antibodies from different patients. As was shown earlier in the blood of patients with certain autoimmune pathologies [32,33] and demonstrated in this article, abzymes are produced that hydrolyze different model RNAs, including homo-oligonucleotides (pA)_23_, (pC)_23_, and (pU)_23_. In addition to Abs against microRNAs, the blood of MS patients may contain abzymes against several other RNAs, which may have partial homology with different fragments of the eight individual miRNAs used by us. In this case, abzymes against other RNAs can hydrolyze the eight microRNAs in other sites, leading to unspecific splitting.

Thus, most probably, the polyclonal IgGs of some patients can contain monoclonal Abs specific for several different miRNAs. At the same time, some IgG preparations hydrolyze miRNAs nonspecifically, similar to the hydrolysis by IgGs of homo-oligonucleotides. Possibly, this may be due to the fact that these polyclonal immunoglobulins do not contain specific IgG-abzymes against these specific miRNAs. At the same time, it should be said that in the blood of a large number of MS patients, specific abzymes against miR-137 may be produced more effectively than against other miRNAs. Moreover, abzymes against miR-137 from sera of all MS patients show the same distinct site-specific recognition and splitting. Thus, the relative effectiveness of the site-specific and nonspecific hydrolysis of individual microRNAs in the case of each of them and each polyclonal preparation of IgGs will depend on the ratio of abzymes against individual microRNA and other various RNAs.

The 14 individual IgGs of conditionally healthy donors possessing RNase activity hydrolyzed different miRNAs. The average value of RAs of the 14 IgG healthy donors demonstrates 22.3–57.3-fold lower average RAs than the Abs of 23 MS patients (Table S6). Differences between RAs of Abs of healthy donors and all four groups of MS patients (CISMS, RRMS, SPMS, and PPMS) were statistically significant; *p*-values varied from 0.0001 to 0.015. The maximum difference in average RAs in the hydrolysis of the eight miRNAs by IgGs of the three main groups of MS patients (CISMS, RRMS, and PPMS) varied from 1.3 to 2.2-fold.

The CCs of RA cohorts corresponding to the hydrolysis of the eight miRNAs in the case of every one of the four groups were very different and varied from positive (+0.95) to negative (−0.31). The reason for this might be that in the case of each individual MS patient, auto-Abs and abzymes against various miRNAs are produced with different efficiencies and to various miRNAs.

An earlier analysis of the relative activity of the hydrolysis of four miRNAs (miR-137, miR-9-5p, miR-219-2-3p, and miR-219a-5p) by 21 IgG preparations from the serum of patients with schizophrenia was carried out [43,44]. Interestingly, the average efficiencies of hydrolysis of four of these miRNAs, with 21 Abs preparations from sera of patients with schizophrenia (average value 61.6 ± 32.7, M = 62.9, IQR = 60.6%) [43,44] and 23 patients with MS (average value 51.2 ± 35.8, M =45.8, IQR =71.3%), were only slightly different. A similar situation for these four miRNAs was observed for 11 IgG preparations of SLE patients (average value 56.0 ± 29.8, M = 56.7, IQR = 30.1%) [66]. At the same time, there are some differences in relative activities in the hydrolysis of some -RNAs by IgGs of patients with MS, SLE, and schizophrenia. The maximum and minimum activity of IgGs in MS patients is observed in the case of miR-155-5p and miR-137, respectively, while it is observed for SLE antibodies for miR-9-5p and miR-219a-5p [66] and for abzymes of schizophrenia patients for miR-219a-5p and miR-219a-2-3p [43,44].

The reason for this may be that these diseases have some similarities, and at the same time, they differ in the relative efficiency of the synthesis of various microRNAs and abzymes against them.

As noted above, dysregulation of several miRNAs is shown in MS [56,57,58]. Therefore, it can be assumed that the hydrolysis of these immunoregulatory miRNAs by IgG antibodies of MS patients may aggravate the dysregulation of these miRNA levels in serum of MS patients. This can lead to a variety of effects since these immunoregulatory miRNAs are involved in multiple pathways in MS patients. For instance, activation of the NFκB pathway induces the expression of miR-146a, which, in turn, inhibits the translation of IRAK1 and TRAF6, key players in this pathway [37]. Another miRNA, miR-21, effectively modulates neuroinflammation through inhibition of Pdcd4 expression, thereby inhibiting the activity of transcription factor NFκB, decreasing IL-10 production and increasing IL-6 [72]. Therefore, a decrease in expression of anti-inflammatory miR-21 or miR-146a contributes to inflammation and autoimmune pathology. On the contrary, the hydrolysis of proinflammatory miRNAs (miR-155-5p and miR-326) can reduce neuroinflammation in MS. The hydrolysis of neuroregulatory miRNAs can also have many consequences. For example, miR-219 and miR-9 are known to promote the early and late stages of oligodendrocyte progenitor cell differentiation, production of myelin, and maintenance of mature myelin sheath [73,74]. Thus, the hydrolysis of these miRNAs by IgGs of patients can disrupt remyelination processes in MS. Although miRNAs are mainly localized in the cell, various miRNAs circulate in the extracellular space and bloodstream as part of exosomes and complexes with proteins or lipoproteins [41]. Interestingly, extracellular miRNA let-7 induces inflammation and neurodegeneration through the activation of RNA-sensing Toll-like receptor 7 [75]. Consequently, antibodies hydrolyzing extracellular miRNAs can compensate for neurodegeneration and inflammation in MS. Therefore, in general, catalytic IgGs may play both a negative and a compensatory role in MS pathogenesis.

It has been shown that similar DNA-, RNA-, and MBP-hydrolyzing Abs, playing an important harmful role in MS pathogenesis (for reviews, see [7,8,9,10,11]), are detected in sera of schizophrenia patients [43,44,45,46,47]. Some similar neuropsychiatric indicators of these diseases are also common for SCZ, SLE, and MS patients [3].

## 4. Materials and methods

### 4.1. Chemicals, Donors, and Patients

Most proteins and chemicals were from Sigma (St. Louis, MO, USA), and the Superdex 200 HR 10/30 column was from GE Healthcare (GE Healthcare Life Sciences, New York, USA). RNase A and FastAP thermosensitive alkaline phosphatase were from Fisher Scientific (Pittsburgh, PA, USA).

Blood samples of patients with MS and healthy donors were obtained from the Neurological Clinic at the Department of Neurosurgery and Neurology of the Siberian State Medical University. The blood serum of 14 healthy volunteers (18–40 years old; average value 28.0 ± 9.0; 7 women and seven men) and 23 MS patients of different ages (20–58 years old; average value 35.8 ± 11.8; 18 women and 5 men) were used for the study. More detailed data on the patients are presented in the Supplementary Materials (Table S1).

The study protocol was checked and approved by the ethics committee of the Novosibirsk State Medical University (Novosibirsk, Russia; the number of permissions—72). In addition, according to the Helsinki ethics committee guidelines, informed consent was obtained from patients to present their blood for scientific purposes.

The diagnosis of MS was made by qualified neurologists in accordance with the recommendations of the 2010 and 2017 revisions according to the McDonald criteria [76,77], including the Expanded Disability Status Scale (EDSS) [78]. The general group of patients (23 individuals) was divided into four different groups: clinically isolated syndrome (CISMS, eight patients); relapsing-remitting MS (RRMS, nine patients); secondary progressive MS (SPMS, five patients); primary progressive MS (PPMS; one patient) (Table S1).

All CISMS patients were at high risk of developing MS because they had MRI signs of demyelination. In the six CISMS patients, the presence of monofocal or multifocal foci was accompanied by severe clinical symptoms, while two CISMS patients had clinically silent brain lesions. However, according to the 2017 revision of the McDonald criteria, such patients cannot be diagnosed with MS because, due to the limited follow-up time, dissemination in time (DIT) or dissemination in space (DIS) by an additional clinical attack, implicating a different CNS site, or by MRI has not been demonstrated.

Upon admission to the clinic, the MS patients had no symptoms of acute infections. However, some of the patients at the time of sample collection had not received any treatment against MS before the study, while others were treated with different drugs, including dexamethasone, glatiramer acetate, interferon β-1b, and natalizumab (Table S1). After passing the mandatory annual medical examination of the institute’s employees, 14 of 300 people, healthy according to various analyzes and the conclusion of doctors of various profiles, were selected for the control study. For comparison, we selected 14 healthy donors with no history of autoimmune, rheumatologic, viral, gastrointestinal, respiratory, cardiovascular, reproductive, or nervous system pathologies.

### 4.2. IgG Purification

Electrophoretically homogeneous IgGs were separated from other proteins, first by affinity chromatography of serum proteins on protein G-sepharose, and then using FPLC gel filtration, as in [13,14,15,16,17,18]. Details of the used methods are given in the Supplementary Materials (IgG purification).

### 4.3. Analysis of Homo-Oligonucleotides and miRNA Hydrolysis by IgGs

Fluorescently labeled (fluorescein isothiocyanate; Flu) homo-oligonucleotides 5’-Flu-(pA)_23_, 5’-Flu-(pC)_23_, 5’-Flu-(pU)_23_, as well as several miRNAs participating in the regulation of neuroinflammation and characterized by impaired expression in MS [37], were used in the study. They are the following four neuroregulatory miRNAs:

miR-137 (5’-Flu-UUAUUGCUUAAGAAUACGCGUAG),
miR-9-5p (5’-Flu-UCUUUGGUUAUCUAGCUGUAUGA),
miR-219-2-3p (5’-Flu-AGAAUUGUGGCUGGACAUCUGU), and
miR-219-5p (5’-Flu- UGAUUGUCCAAACGCAAUUCU)

as well as the following immunoregulatory ones:

miR-21-3p (5′- Flu-CAACACCAGUCGAUGGGCUGU),
miR-146a-3p (5′-Flu-CCUCUGAAAUUCAGUUCUUCAG),
miR-155-5p (5′- Flu-UUAAUGCUAAUCGUGAUAGGGGU), and
miR-326 (5′- Flu-CCUCUGGGCCCUUCCUCCAG).

The reaction mixture (10 μL) contained 50 mM Tris-HCl (pH 7.5), 0.01 mg/mL of one of miRNAs (depending on the miRNA, 1.3–1.6 μM was used), and 0.6 µM IgGs. It was incubated for 1 h at 37 °C. The reaction was stopped by the addition of a denaturing buffer (10 μL) consisting of 8 M urea and 0.025% xylene cyanol. The molecular weights of the RNA markers were obtained by 3.2 μM miRNAs statistical alkaline hydrolysis (at all internucleoside bonds) by their incubation in 50 mM bicarbonate buffer, pH 9.5, for 15 min at 95 °C.

For comparison of thermal stability of IgGs and RNase A, they were incubated for 15 min at 30, 40, 50, 70, 80, and 100 °C, and their relative activity was analyzed, as described above, using Tris-HCl, pH 7.5, and miR-137 as the substrate.

### 4.4. Application of Strict Criteria

An equimolar mixture of 23 preparations of IgGs (IgG_mix_) was incubated in 50 mM glycine-HCl buffer (pH 2.6) containing 0.4 M NaCl for 30 min at 25 °C. Separation of the IgG_mix_ under conditions of “acid shock” was carried out by FPLC gel filtration on a Superdex 200 column equilibrated with 30 mM glycine-HCl (pH 2.6), supplemented with 0.1 M NaCl, as in [13,14,15,16,17]. Fractions were collected, then neutralized and sterilized, as described above. After one week of storage (4 °C) for refolding after the acid shock, IgG preparations were used for the RNase activity assay, as described above.

IgG_mix_ was also chromatographed on anti-IgG-sepharose bearing immobilized mouse Abs against human IgGs, as in [13,14,15,16,17]. The IgG_mix_ preparation was applied on a 1-mL column equilibrated with 25 mM Tris-HCl (pH 7.5), supplemented with 0.1 M NaCl, and the column was then washed with the same buffer containing 0.3 M NaCl. IgGs were specifically eluted using 0.1 M glycine-HCl (pH 2.6), neutralized, dialyzed, sterilized, and used for assay of RNase activity, as described above.

### 4.5. Determination of the Kinetic Parameters

We have estimated the *K*_m_ values for several microRNAs in their hydrolysis with IgGs, according to [79]. The dependencies of the initial rates on the microRNA concentrations in the hydrolysis reaction were consistent with Michaelis–Menten kinetics. The *K*_m_ values were determined.

### 4.6. Statistical Analysis

To check for normality of value distribution, Shapiro–Wilk’s W Test criterion was used. The activity of most of the sample sets did not meet the normal Gaussian distribution. The nonparametric ranking method of Spearman was mainly used for correlation analysis. In the case where the data obeyed normal distribution, the parametric method of Pearson was used. To evaluate the differences between the samples, the Mann–Whitney U test was used; *p* < 0.05 was considered statistically significant. The median (M) and interquartile ranges (IQRs) were estimated.

## 5. Conclusion

In this work, we first demonstrate that the blood of patients with multiple sclerosis contains antibodies against miRNAs that recognize and catalyze the site-specific hydrolysis of eight miRNAs efficiently. However, one cannot exclude that miRNA-, MBP-, and DNA-hydrolyzing abzymes, in addition to other different factors, may cooperatively promote important neuropathologic mechanisms in MS and SCZ pathogenesis.

## Figures and Tables

**Table 1:** Average relative activities (%) of polyclonal IgGs from the sera of MS patients and conditionally healthy donors in the hydrolysis of eight different microRNAs*.

Groups of Patients and Healthy Donors	miR-137	miR-9-5p	miR-219-2-3p	miR-219-5p	miR-21-3p	miR-146a-3p	miR-155-5p	miR-326
**Clinically isolated syndrome MS (CISMS, 8 patients)**								
Mean ± SD for individual RNAs **	**65.4 ± 27.2**	**40.7 ± 35.8**	**26.9 ± 30.2**	**25.6 ± 29.6**	**46.8 ± 28.6**	**50.6 ± 32.6**	**69.4 ± 17.3**	**33.6 ± 28.7**
Median (IQR) for individual RNAs ***	**64.9 (36.9)**	**24.0 (62.0)**	**12.5 (44.2)**	**14.0 (31.6)**	**38.7 (39.8)**	**48.6 (53.4)**	**68.5 (27.1)**	**24.6 (27.9)**
**Relapsing-remitting MS (RRMS. 9 patients)**								
**Mean ± SD for individual RNAs**	**33.3 ± 31.4**	**54.9 ± 39.7**	**55.3 ± 39.7**	**55.2 ± 32.2**	**59.8 ± 26.4**	**65.0 ± 33.4**	**83.0 ± 13.6**	**45.0 ± 33.4**
**Median (IQR) for individual RNAs**	**21.6 (11.9)**	**41.2 (75.8)**	**38.8 (76.7)**	**67.8 (50.8)**	**68.6 (38.7)**	**63.0 (49.8)**	**80.0 (22.8)**	**36.9 (55.4)**
**Primary progressive MS (PPMS, 1 patient)**								
**Mean for individual RNAs**	**50.4**	**98.1**	**96.4**	**93.2**	**98.7**	**89.6**	**97.9**	**94.0**
	**Secondary progressive MS (SPMS, 5 patients)**							
**Mean ± SD for individual RNAs**	**39.8 ± 51.7**	**42.9 ± 50.2**	**43.7 ± 48.1**	**52.0 ± 44.9**	**45.1 ± 46.1**	**44.5 ± 47.5**	**49.2 ± 39.4**	**33.3 ± 31.4**
**Median (IQR) for individual RNAs**	**3.4 (91.9)**	**12.2 (91.6)**	**20.8 (90.4)**	**50.8 (86.5)**	**14.0 (81.6)**	**14.7 (85.5)**	**24.2 (66.1)**	**6.6 (91.5)**
	**Conditionally healthy donors (14 volunteers)**							
**Mean ± SD for individual RNAs**	**1.2 ± 2.3**	**3.9 ± 2.7**	**1.7 ± 2.7**	**1.6 ± 2.4**	**1.5 ± 1.8**	**2.6 ± 3.0**	**2.8 ± 2.9**	**3.9 ± 3.3**
Median (IQR) for individual RNAs **	**0 (2.3)**	**4.2 (3.6)**	**0 (4.6)**	**0.4 (3.3)**	**1.0 (3.0)**	**2.5 (4.5)**	**3.4 (6.5)**	**0 (2.7)**

* Summary of the main data given in Tables S2–S6. ** The average values of RAs characterizing hydrolysis of individual RNAs by several IgGs corresponding to each group of patients. *** The median (M) and interquartile ranges (IQRs) characterizing hydrolysis of individual RNAs by several IgGs corresponding to each group of patients.

**Table 2:** Average relative activities of polyclonal IgGs from the sera of MS patients and conditionally healthy donors in the hydrolysis of eight different microRNAs by IgGs of different groups were ranked from the highest (digit 1) to lowest (digit 8) activity (more detailed data are given in Table S6).

Group MS Patients and Healthy Humans	Different microRNAs							
	miR-137	miR-9-5p	miR-219-2-3p	miR-219-5p	miR-21-3p	miR-146a-3p	miR-155-5p	miR-326
	Conventional Units of Relative Activity from 1 (Maximum Activity) to 8 (Minimum Activity)*							
CISMS, 8 patients	**2**	**5**	**7**	**8**	**4**	**3**	**1**	**6**
RRMS. 9 patients	**8**	**6**	**5**	**4**	**3**	**2**	**1**	**7**
PPMS. 1 patient	**8**	**2**	**4**	**6**	**1**	**7**	**3**	**5**
SPMS, 5 patients	**7**	**6**	**5**	**1**	**3**	**4**	**2**	**8**
Conditionally healthy donors (14 volunteers)	**8**	**1**	**5**	**6**	**7**	**4**	**3**	**2**
Average values	6.6	4	5.2	5.0	3.6	4	2	5.6

* The average values of RAs characterizing hydrolysis of individual RNAs by several IgGs corresponding to four groups of MS patients and healthy donors.

## Data Availability

All of the data that were generated and analyzed in this study are included in this article and the Supplementary Materials.

## Supplementary Materials

The supplementary materials can be found at https://www.mdpi.com/1422-0067/22/6/2812/s1. Table S1 (clinical data for multiple sclerosis patients included in the study); Tables S2–S6 (data on the relative activity of IgGs in the hydrolysis of eight microRNAs).

## Abbreviations

Abs, antibodies; Abzs, abzymes or catalytic antibodies; AI, autoimmune; AIDs, autoimmune diseases; MS, multiple sclerosis; CISMS, clinically isolated syndrome of MS; EDTA, ethylenediaminetetraacetic acid; PAGE, polyacrylamide gel electrophoresis; PPMS, primary progressive MS; RA, relative activity; RRMS, relapsing-remitting MS; SLE, systemic lupus erythematosus, SDS, sodium dodecyl sulfate; SPMS, secondary progressive MS.
