# Supplementary material for: IgGs-Abzymes from the Sera of Patients with Multiple Sclerosis Recognize and Hydrolyze miRNAs
Source: Int J Mol Sci. 2021 Mar 10;22(6):2812. doi: 10.3390/ijms22062812 (PMC8000798; doi:10.3390/ijms22062812)
Supplement: Supplementary file 1 [file ijms-22-02812-s001.pdf]

Supplementary methods and Tables

IgG purification

IgG purification was carried as in [13-18]. The serum samples (2 ml) were applied onto protein G-Sepharose columns (1 ml) equilibrated in buffer A (50 mM Tris-HCl, pH 7.5, 150 mM NaCl). The columns were then washed using buffer A (7 ml) to zero optical density (A_{280}). Non-specifically bound proteins were additionally removed with 4 ml buffer A containing 0.5 M NaCl and 1% Triton X-100. Then the columns were washed with buffer A containing no Triton X-100 to zero optical density. IgGs were eluted specifically with glycine-HCl buffer (0.1 M; pH 2.6); the fractions were collected in cooled tubes containing 50 µl of 1.0 M Tris-HCl (pH 8.5). All fractions were then neutralized using this buffer and dialyzed against 50 mM Tris-HCl containing 50 mM NaCl (pH 7.5). The IgGs fractions of the central part of the antibodies peak were merged together and concentrated for FPLC gel filtration. Before FPLC gel filtration, Abs were preincubated in 40 mM glycine-HCl buffer (pH 2.6) at 25° C for 12 min. Purification of the IgGs was performed by FPLC gel filtration using a column of Superdex 200 HR 10/30 equilibrated with 10 mM glycine HCl (pH 2.6) containing 0.1 M NaCl as in [14-16,43,44,53,55]. Fractions were collected, neutralized and sterilized using filtration through 0.2 µm Millex filter. After 2 weeks of storage at 4° C for refolding of IgGs after their acid shock (when Abs were used later they were frozen at – 70°C), the IgGs were used for the activity assays as described below.

References [13-18] are given in the article text:

13. Polosukhina, D.I.; Kanyshkova, T.G.; Doronin, B.M.; Tyshkevich, O.B.; Buneva, V.N.; Boiko, A.N. Gusev, E.I., Favorova, O.O.; Nevinsky, G.A. Hydrolysis of myelin basic protein by polyclonal catalytic IgGs from the sera of patients with multiple sclerosis. *J. Cell Mol. Med.* **2004**, *8*, 359–368.

14. Polosukhina, D.I.; Kanyshkova, T.G.; Doronin, B.M.; Tyshkevich, O.B.; Buneva, V.N.; Boiko, A.N.; Gusev, E.I.; Nevinsky, G.A.; Favorova, O.O. Metal-dependent hydrolysis of myelin basic protein by IgGs from the sera of patients with multiple sclerosis. *Immunol. Lett*. **2006**, *103*, 75–81.

15. Legostaeva, G. A.; Polosukhina, D.I.; Bezuglova, A.M.; Doronin, B.M.; Buneva, V.N.; Nevinsky, G.A. Affinity and catalytic heterogeneity of polyclonal myelin basic protein-hydrolyzing IgGs from sera of patients with multiple sclerosis. *J. Cell Mol. Med*. **2010**, *14*, 699-709.

16. Bezuglova, A.V.; Konenkova, L.P.; Doronin, B.M.; Buneva, V.N.; Nevinsky, G.A. Affinity and catalytic heterogeneity and metal-dependence of polyclonal myelin basic protein-hydrolyzing IgGs from sera of patients with systemic lupus erythematosus. *J. Mol. Recognit.* **2011**, *24*, 960–974.

17. Baranovskii, A.G.; Kanyshkova, T.G.; Mogelnitskii, A.S.; Naumov, V.A.; Buneva, V.N.; Gusev, E. I.; Boiko, A.N.; Zargarova, T.A.; Favorova, O.O.; Nevinsky, G.A. Polyclonal antibodies from blood and cerebrospinal fluid of patients with multiple sclerosis effectively hydrolyze DNA and RNA. *Biochemistry (Mosc)* **1998**, *63*,1239-1248.

18. Baranovskii, A.G.; Ershova, N.A.; Buneva, V.N.; Kanyshkova, T.G.; Mogelnitskii, A.S.; Doronin, B.M.; Boiko, A.N.; Gusev, E.I.; Favorova, O.O.; Nevinsky, G.A. Catalytic heterogeneity of polyclonal DNA-hydrolyzing antibodies from the sera of patients with multiple sclerosis. *Immunol. Lett*. **2001**, *76*, 163-167.

.

Supplementary Table S1. Clinical characteristics of MS patients

N	Course of MS*	Sex	Age (years)	Disease duration (years)	Prevailing symptoms at debut[α]	Disease stage	EDSS steps[β]	Therapy
Clinically isolated syndrome (CISMS)								
1	CIS	F	26	0	RBN	Exacerbation	3.0	Dexamethasone
2	CIS	F	24	0.2	OI	Exacerbation	4.5	Dexamethasone
3	CIS	F	21	0	RBN	Exacerbation	2.0	Dexamethasone
4	CIS	M	30	0	PSI	Exacerbation	3.5	Dexamethasone
5	CIS	F	29	0	PSI	Exacerbation	3.5	Dexamethasone
6	CIS	F	20	0	N.D.	Exacerbation	2.0	Without treatment
7	CIS	F	26	0	N.D.	Exacerbation	2.0	Without treatment
8	CIS	F	43	0	PSI	Exacerbation	4.0	Dexamethasone
Average values	8 patients	7 F/ 1 M	27.4±7.2	0.02±0.07	3 PSI/ 2 RBN/ 1 OI/ 2 N.D.	8 Ex.	3.1±0.9	6 Dex./ 2 W. tr.
Relapsing-remitting multiple sclerosis (RRMS)								
9	RRMS	F	23	3	RBN	Remission	2	Glatiramer acetate
10	RRMS	F	37	6	SSI	Remission	3	Glatiramer acetate
11	RRMS	F	38	12	SSI	Exacerbation	3	Interferon β-1b
12	RRMS	F	45	11	SSI	Exacerbation	4	Glatiramer acetate
13	RRMS	M	52	3	PSI	Remission	2	Glatiramer acetate
14	RRMS	F	21	0,25	CI	Exacerbation	1.5	Dexamethasone
15	RRMS	M	46	16	SSI	Exacerbation	4.0	Dexamethasone
16	RRMS	F	22	1	PSI	Exacerbation	3.5	Natalizumab
17	RRMS	F	45	1	N.D.	Exacerbation	3.0	Dexamethasone
Average values	9 patients	7 F/ 2 M	36.6±11.8	5.9±5.7	4 SSI/ 2 PSI/ 1 RBN/ 1 CI/ 1 N.D.	6 Ex./ 3 Rem.	2.9±0.9	4 G.a./ 3 Dex./ 1 Inf./ 1 Nat.
Primary progressive multiple sclerosis (PPMS)								
18	PPMS	F	45	0	PSI	Exacerbation	7.0	Dexamethasone
Average values	1 patient	1 F	45	0	1 PSI	1 Ex.	7.0	1 Dex.
Secondary progressive multiple sclerosis (SPMS)								
19	SPMS	F	53	23	SSI	Remission	3.5	Interferon β-1b
20	SPMS	F	58	20	PSI	Remission	3	Interferon β-1b
21	SPMS	M	38	9	PSI	Remission	4	Interferon β-1b
22	SPMS	M	33	10	PSI	Exacerbation	3.5	Glatiramer acetate
23	SPMS	F	48	17	PSI	Remission	4.5	Interferon β-1b
Average values	5 patients	3 F/ 2 M	46±10.4	15.8±6.1	4 PSI/ 1 SSI	1 Ex./ 4 Rem.	3.7±0.6	4 Inf./ 1 G.a.
Average values for total	23 patients	18 F/ 5 M	35.8±11.8	5.8±7.4	10 PSI/ 5 SSI/ 3 RBN/	16 Ex./ 7 Rem.	3.3±1.1	10 Dex./ 5 G.a./ 5 Inf./

group					1 CI/<br>1 OI/<br>3 N.D.			1 Nat./<br>2 W. tr.

* In accordance with the recommendations of the 2010 and 2017 revisions of the McDonald criteria (Polman, C. H., Reingold, S. C., Banwell, B., Clanet, M., et al. (2011). Diagnostic criteria for multiple sclerosis: 2010 revisions to the McDonald criteria. Annals of neurology, 69(2), 292-302; Thompson, A. J., Banwell, B. L., Barkhof, F., Carroll, W. M., et al. (2018). Diagnosis of multiple sclerosis: 2017 revisions of the McDonald criteria. The Lancet Neurology, 17(2), 162-173).

[α] Abbreviations for prevailing symptoms at debut: RBN - retrobulbar neuritis, SSI - sensory system impairments, OI - oculomotor impairments, CI - cerebellar impairments, PSI - pyramidal system impairments, N.D. – no data.

[β] Based on the Kurtzke Expanded Disability Status Scale (EDSS) (Kurtzke, J. F. (1983). Rating neurologic impairment in multiple sclerosis: an expanded disability status scale (EDSS). Neurology, 33(11), 1444-1444).

Supplementary Table S2. Relative hydrolysis of eight different miRNAs by polyclonal IgGs from the sera of 8 MS patients with the clinically isolated syndrome.

Numbers of patients and corresponding IgG preparations	miRNA-hydrolyzing activity of IgG, %*							
	miR-137 (1)	miR-9-5p (2)	miR-219-2-3p (3)	miR-219-5p (4)	miR-21-3p (5)	miR-146a-3p (6)	miR-155-5p (7)	miR-326 (8)
Clinically isolated syndrome (CISMS)								
1	78.3	62.0	17.3	30.7	38.3	81.8	50.2	20.0
2	80.2	23.2	4.5	13.3	33.3	18.3	62.2	57.7
3	98.2	94.2	83.8	42.8	98.0	98.2	96.8	93.8
4	18.5	18.2	3.1	6.7	39.1	63.4	77.1	26.8
5	51.5	5.4	7.7	3.6	15.0	8.5	74.9	7.1
6	50.7	8.7	2.6	2.5	19.0	28.7	48.3	11.2
7	95.2	88.9	39.3	90.3	54.6	72.1	59.2	29.4
8	50.9	24.8	56.7	14.7	77.3	33.9	86.5	22.5
Coeff. correlation between parameters 1-8	1-2, 2-4, 2-6, and 3-5 were in range +0.81-0.95; 1-4, 2-3, 3-7, 5-7, and 5-8 – +0.68-0.72; 1-3. 1-8, 2-8, 3-6, 3-8, 4-6, 5-6 – + 0.54-0.63; 1-5, 1-6, 3-4, 4-5, 6-8, 7-8 - + 0.43-0.59; for 2-7 - +017; 6-7 - +0.12, 1-7 - -0.04, and 2-7 - - 0.05							
Mean ± SD for individual RNAs	65.4 ± 27.2	40.7 ± 35.8	26.9 ± 30.2	25.6 ± 29.6	46.8 ± 28.6	50.6 ± 32.6	69.4 ± 17.3	33.6 ± 28.7
Median (IQR) for individual RNAs**	64.9 (36.9)	24.0 (62.0)	12.5 (44.2)	14.0 (31.6)	38.7 (39.8)	48.6 (53.4)	68.5 (27.1)	24.6 (27.9)
The average value for all microRNAs	44.9 ± 31.6							
M (IQR) for all microRNAs	39.2 (58.2)							

*For each value, a mean of 3 measurements is reported; the error of the determination of values did not exceed 7-10 %.

**The median (M) and interquartile ranges (IQR).

Supplementary Table S3. **Relative hydrolysis of eight different microRNAs by polyclonal IgGs from the sera of 9 MS patients with relapsing-remitting multiple sclerosis (RRMS).**

Numbers of patient and corresponding IgG preparations	miRNA-hydrolyzing activity of IgG, %*							
	miR-137 (1)	miR-9-5p (2)	miR-219-2-3p (3)	miR-219-5p (4)	miR-21-3p (5)	miR-146a-3p (6)	miR-155-5p (7)	miR-326 (8)
Relapsing-remitting multiple sclerosis (RRMS)								
9	21.6	24.5	5.9	7.4	22.4	15.1	74.7	11.6
10	79.8	94.2	92.4	23.0	95.6	98.3	79.1	36.9
11	15.1	12.7	32.4	67.8	79.1	54.8	98.5	26.0
12	13.2	13.9	19.9	45.1	22.9	19.0	59.3	17.9
13	17.0	98.2	96.7	85.8	52.9	63.0	81.8	86.1
14	94.2	93.3	97.7	69.0	79.2	90.3	99.3	8.2
15	26.5	97.7	96.9	89.2	68.6	98.2	99.0	98.0
16	27.0	18.4	38.8	8.8	76.7	98.2	80.0	73.3
17	5.4	41.2	16.8	73.8	40.4	48.4	75.7	46.6
Coeff. correlation between parameters 1-8	Parameters 2-3, 5-6, and 3-6 - +0.88-0.93; 1-3, 1-5, 2-6, 3-5, 5-7, and 6-7 - + 0.6-0.66; 1-6, 3-7, 6-8 - +0.5-0.58; 2-4, 2-5, 2-7, 3-4, 3-8, 4-7 - + 0.44-0.49; 1-7, 2-8, 4-8 – + 0.36-0.39; 5-8, and 7-8 - + 0.21; 4-5 - +0.07; 4-6 - +0.14, 1-4 - - 0.13, 1-8 - -0.31							
Mean ± SD for individual RNAs	33.3 ± 31.4	54.9 ± 39.7	55.3 ± 39.7	55.2 ± 32.2	59.8 ± 26.4	65.0 ± 33.4	83.0 ± 13.6	45.0 ± 33.4
Median (IQR) for individual RNAs**	21.6 (11.9)	41.2 (75.8)	38.8 (76.7)	67.8 (50.8)	68.6 (38.7)	63.0 (49.8)	80.0 (22.8)	36.9 (55.4)
Average value for all microRNAs	56.1 ± 33.5							
M (IQR) for all microRNAs	61.1 (67.7)							

*For each value, the average value of three measurements is reported; the error of the determination of the values did not exceed 7-10 %.

**The median (M) and interquartile ranges (IQR).

Supplementary Table S4. Relative hydrolysis of eight different miRNAs by polyclonal IgGs from the sera of 1 MS patients with primary progressive multiple sclerosis, five with secondary progressive multiple sclerosis.

Numbers of the patient and corresponding IgG preparations	miRNA-hydrolyzing activity of IgG, %*							
	miR-137 (1)	miR-9-5p (2)	miR-219-2-3p (3)	miR-219-5p (4)	miR-21-3p (5)	miR-146a-3p (6)	miR-155-5p (7)	miR-326 (8)
Primary progressive multiple sclerosis (PPMS)								
18	50.4	98.1	96.4	93.2	98.7	89.6	97.9	94.0
The average value for all microRNA	89.8 ± 16.2							
M (IQR) for all microRNAs	95.2 (6.6)							
Secondary progressive multiple sclerosis (SPMS)$^{\alpha}$								
19	1.2	5.6	2.2	9.8	14.0	9.7	20.2	2.9
20	99.3	97.2	94.2	96.3	96.7	95.2	97.8	95.5
21	93.4	98.1	97.4	98.0	94.3	97.5	86.3	96.2
22	3.4	12.2	3.8	50.8	12.7	14.7	24.2	6.6
23	1.5	1.2	20.8	5.0	7.8	5.3	17.7	4.0
Coefficient correlation	All CCs in the range 0.87-0.99							
Mean ± SD for individual RNAs	39.8 ± 51.7	42.9 ± 50.2	43.7 ± 48.1	52.0 ± 44.9	45.1 ± 46.1	44.5 ± 47.5	49.2 ± 39.4	33.3 ± 31.4
Median (IQR) for individual RNAs**	3.4 (91.9)	12.2 (91.6)	20.8 (90.4)	50.8 (86.5)	14.0 (81.6)	14.7 (85.5)	24.2 (66.1)	6.6 (91.5)
The average value for all microRNAs	44.8 ± 43.1							
M (IQR) for all microRNAs	18.9 (90.4)							

*For each value analyzed, a mean of three measurements is reported; the error of the determination of values did not exceed 7-10 %.

**The median (M) and interquartile ranges (IQR).

Supplementary Table S5. Relative hydrolysis of eight different miRNAs by polyclonal IgGs from the sera of 14 conditionally healthy donors.

Numbers of patient and corresponding IgG preparations	miRNA-hydrolyzing activity of IgG, %*							
	miR-137 (1)	miR-9-5p (2)	miR-219-2-3p (3)	miR-219-5p (4)	miR-21-3p (5)	miR-146a-3p (6)	miR-155-5p (7)	miR-326 (8)
Healthy donors								
1	7.8	6.4	0	0	7.3	8.8	9.4	0
2	0	2.9	5.2	2.7	0	0	3.1	3.4
3	4.4	5.7	0	0	5.1	4.5	3.8	0
4	0	3.6	5.1	4.3	0	5.8	7.2	0
5	2.2	4.8	5.7	3	3.3	4.4	0	4.6
6	0	5.5	2.7	3.8	7.1	4.2	3.1	0
7	0.7	8.2	4.6	3.8	5.4	5.5	9.7	4.3
8	1.3	6.9	0.3	3.3	6.4	4.2	2.8	2.7
9	0	5.2	0	0.8	1.1	0.7	6.5	0
10	0	3.2	0	0	0	0.8	4.7	0
11	0	2.1	0	0	0.9	0.4	4.2	0
12	0	0	0	0	0	0	0	0
13	0	0	0	0	0	0	0	0
14	0	0	0	0	0	0	0	0
Mean ± SD for individual RNAs	1.2 ± 2.3	3.9 ± 2.7	1.7 ± 2.7	1.6 ± 2.4	1.5 ± 1.8	2.6 ± 3.0	2.8 ± 2.9	3.9 ± 3.3
Median (IQR) for individual RNAs**	0 (2.3)	4.2 (3.6)	0 (4.6)	0.4 (3.3)	1.0 (3.0)	2.5 (4.5)	3.4 (6.5)	0 (2.7)
The average value for all microRNAs	2.3 ± 2.7							
M (IQR) for all microRNAs	0.8 (4.4)							
Spearman's corr. coefficient	The correlation coefficient (R) varied from +0.11 to +0.85.							

*For each value, the avarage value of three measurements is reported; the error of the determination of values did not exceed 7-10 %.

**The median (M) and interquartile ranges (IQR)

Supplementary Table S6. **Average relative activities of polyclonal IgGs from the sera of MS patients and conditionally healthy donors in the hydrolysis of eight different microRNAs.**

Group of patients and healthy donors	miR-137 (1)	miR-9-5p (2)	miR-219-2-3p (3)	miR-219-5p (4)	miR-21-3p (5)	miR-146a-3p (6)	miR-155-5p (7)	miR-326 (8)
Clinically isolated syndrome MS (CISMS, 8 patients)								
Mean ± SD for individual RNAs*	65.4 ± 27.2	40.7 ± 35.8	26.9 ± 30.2	25.6 ± 29.6	46.8 ± 28.6	50.6 ± 32.6	69.4 ± 17.3	33.6 ± 28.7
Median (IQR) for individual RNAs**	64.9 (36.9)	24.0 (62.0)	12.5 (44.2)	14.0 (31.6)	38.7 (39.8)	48.6 (53.4)	68.5 (27.1)	24.6 (27.9)
Order of relative RA$^{\Omega\iota}$	2	5	7	8	4	3	1	6
The average value for all microRNAs***	44.9 ± 31.6							
M (IQR) for all microRNAs***	39.2 (58.2)							
Relapsing-remitting MS (RRMS. 9 patients)								
Mean ± SD for individual RNAs	33.3 ± 31.4	54.9 ± 39.7	55.3 ± 39.7	55.2 ± 32.2	59.8 ± 26.4	65.0 ± 33.4	83.0 ± 13.6	45.0 ± 33.4
Median (IQR) for individual RNAs**	21.6 (11.9)	41.2 (75.8)	38.8 (76.7)	67.8 (50.8)	68.6 (38.7)	63.0 (49.8)	80.0 (22.8)	36.9 (55.4)
Order of relativeRAs$^{\Omega\iota}$	8	6	5	4	3	~2	1	7
The average value for all microRNAs	56.1 ± 33.5							
M (IQR) for all microRNAs	61.1 (67.7))							
Primary progressive MS (PPMS. 1 patient)								
Mean for individual RNAs	50.4	98.1	96.4	93.2	98.7	89.6	97.9	94.0
Order of relative RA	8	2	4	6	1	7	3	5
The average value for all microRNAs	89.8 ± 16.2							
M (IQR) for all microRNAs	95.2 (6.6)							
	Secondary progressive MS (SPMS, 5 patients)							
Mean ± SD for individual RNAs	39.8 ± 51.7	42.9 ± 50.2	43.7 ± 48.1	52.0 ± 44.9	45.1 ± 46.1	44.5 ± 47.5	49.2 ± 39.4	33.3 ± 31.4
Median (IQR) for individual RNAs**	3.4 (91.9)	12.2 (91.6)	20.8 (90.4)	50.8 (86.5)	14.0 (81.6)	14.7 (85.5)	24.2 (66.1)	6.6 (91.5)
Order of relative RA	7	6	5	1	3	4	2	8
The average value for all microRNAs	44.8 ± 43.							
M (IQR) for all microRNAs	18.9 (90.4)							
	Average value 51.2 ± 35.8; median (IQR)) = 45.8 (71.3) for all eight microRNAs,							
	Conditionally healthy donors (14 volunteers)							
Mean ± SD for individual RNAs	1.2 ± 2.3	3.9 ± 2.7	1.7 ± 2.7	1.6 ± 2.4	1.5 ± 1.8	2.6 ± 3.0	2.8 ± 2.9	3.9 ± 3.3
Median (IQR) for individual	0 (2.3)	4.2 (3.6)	0 (4.6)	0.4 (3.3)	1.0 (3.0)	2.5 (4.5)	3.4 (6.5)	0 (2.7)

RNAs**

**Order of relative RA**	**8**	**1**	**5**	**6**	**7**	**4**	**3**	**2**
**The average value for all microRNAs**	**2.3 ± 2.7**							
**M (IQR) for all microRNAs**	**0.8 (4.4)**							

[*]The average values of RAs characterizing hydrolysis of individual RNAs by several IgGs corresponding to each group of patients.

[**]The median (M) and interquartile ranges (IQR) characterizing hydrolysis of individual RNAs by several IgGs corresponding to each group of patients.

[Ωι] Average relative activities of polyclonal IgGs from the sera of MS patients and conditionally healthy donors in the hydrolysis of eight different microRNAs by IgGs of different groups were ranked from the highest (digit 1) to lowest (digit 8) activity

*****Average values, median (M), and interquartile ranges (IQR) for all 8 microRNAs are given.**
